# Beauty centers: an overview of their biosafety conditions in Valledupar, Colombia

**DOI:** 10.47626/1679-4435-2020-586

**Published:** 2021-02-11

**Authors:** Darwin Jose Mendoza, Carlos Mario Zambrano, Emir Rueda

**Affiliations:** 1 Ingenieria Industrial, Universidad de Santander Udes, - Valledupar (Cesar), Colombia; 2 Bacteriologia y Laboratorio Clínico, Universidad de Santander Udes - Valledupar (Cesar), Colombia

**Keywords:** containment of biohazards, beauty and aesthetics centers, occupational health, procedures

## Abstract

**Introduction:**

The risk of transmission of microorganisms in aesthetic and beauty centers is high when mitigation measures are not adopted; thus, it is necessary to constantly review the conditions of these centers, in order to prevent diseases and make the corresponding decisions.

**Objectives:**

To assess biosafety conditions of beauty centers in Valledupar, Colombia.

**Methods:**

This study followed a descriptive methodology and was based on the administration of a survey focused on determining which services are provided in beauty centers, on which activities are developed by their staff, and the conditions under which services are provided.

**Results:**

Study results that 93% of centers are legally constituted; furthermore, the most commonly provided service was hairdressing, with 21%. Only 9% of workers were covered by a social insurance system. In terms of social insurances, centers did not comply with minimum requirements.

**Conclusions:**

The aesthetic centers in Valledupar are not complying with requirements from biosafety protocols set forth by legislation and are thus adopting inadequate procedures.

## INTRODUCTION

The objective of biosafety in establishments that develop cosmetic activities or with purposes of facial, hair, bodily, and ornamental beatification is preventing disease transmission and controlling vectors of transmission, in order to minimize the risk of transfer of pathogens and thus to protect and prevent detrimental impacts. This ensures that the development or the final product of all procedures do not endanger the health and the safety of individuals who work in the field of facial, bodily, and ornamental aesthetics, of their respective work places, of users, and of the environment.^^1^^

The beauty market has considerably grown in recent years, mostly due to media advertising, which has led to the development of techniques that often involves handling of tissues of hands and feet. This practice increases the risk for biological factors underlying diseases, especially blood-related ones.^^2^^

Disease transmission is directly related to the use of personal protection items and to the adoption of appropriate biosafety techniques, in addition to considering factors like the instruments used, since they pose a high risk of contamination.^^3^^

With regard to the use of personal protection equipment, a study assessing beauty centers in Belo Horizonte, Brazil, revealed that 71.5% of the participants did not use personal protection equipment while attending clients.^^4^^ Similarly, another study^^5^^ concluded that manicures and pedicures in Maringá, Brazil, were aware of the appropriate procedures to ensure personal protection, but this knowledge is not appropriately applied in their labor routine. Furthermore, Felipe et al.^^6^^showed that most professionals of the beauty segment who did not wear gloves when they had direct contact with blood used some type of medication or alcohol as preventive measures.

This investigation aimed to assess the biosafety conditions of beauty centers in Valledupar, revealing whether these centers are complying with procedures and processes that ensure the prevention and mitigation of risk factors, especially biological ones. Therefore, an instrument was administered in order to investigate the status of these centers is terms of administration, labor functions, and biosafety to determine whether they worked under conditions that prevent infection transmission.

## METHODS

This project focused on analyzed biosafety conditions in beauty and esthetic centers in Valledupar, for which the finite population formula^^7^^ with correlation factor was applied, and a randomly selected sample of 77 companies was obtained. Instrument administration was accompanied by an informed consent grounded on the following ethical considerations: respect for people and their autonomy; beneficence, i.e., maximizing benefits and minimizing risks for research subjects; and non-maleficence, i.e., avoid doing harm to research subjects.

This study consisted of a descriptive research and was based on the conduction of a survey that sought to describe population characteristics and some of their points of views. Instrument administration was based on the addresses available in the database provided by the regional Chamber of Commerce. However, the centers selected for the survey did not corresponded to the available address; therefore, it was decided to select nearby facilities, but some of these refused to participate in the survey and the sample was thus reduced to 57 centers.

The instruments were administered to randomly selected individuals or to those assigned by the manager or owner of the establishment; in some cases, it was directly administered to those who were administratively responsible for the business. The two questionnaires were primarily focused on determining which services are provided in beauty centers. The first instrument was divided into five sections: the first was related to legal foundations required from the business; the second one focused on working hours and income; the third, on facility’s infrastructure related to walls, ground, etc.; the fourth, on internal information related to conditions facilities have in each process; and, finally, the fifth on some biosafety elements that should be implemented according to current regulations (Table 1).

**Table 1:** Current regulations for aesthetic and beauty centers.

Regulation	Objective	Author*
Law 9 of 1979	National Health Code	8
Decree 2676 of 2000	Comprehensive management of hospital and similar waste	9
Law 711 of 2001	Regulates the exercise of the cosmetology profession	10
Resolution 2117 of 2010	Opening and functioning of ornamentation establishments	11
Resolution 1164 of 2002	Adopts a procedure manual for the comprehensive management of hospital and similar wastes	12
Resolution 2263 of 2004	Opening and functioning of aesthetic centers	13
Resolution 797 of 2004	Regulates decision 516 on control and health surveillance of cosmetics	14
Resolution 2827 of 2006	Adopts biosafety manual	1
Resolution 3924 of 2005	Guide of inspection and opening of functioning of aesthetic centers	15
Colombian Technical Guide (GTC 24)	Colombian technical guide on the separation of sources of waste	16

* According to reference number.

The second instrument focused on determining which functions were performed by the staff, professional’s health status from the labor context, and what personal protection items are required for the appropriate functioning of the activity performed by the professional. Analysis of information was performed using frequencies and means, and data were analyzed using the Epi Info software.

## RESULTS

### CHARACTERIZATION OF SERVICES PROVIDED BY AESTHETIC AND BEAUTY CENTERS

First of all, it was found that 93% of companies have an updated chamber of commerce certificate, implying that these companies comply with the legal process and are subjected to the actions that this entity performs for the benefit of its business structure.

With regard to knowledge on the current regulations, 83% of respondents reported being informed about legislation, i.e., they are aware of what they should implement and develop in order to be in accordance with current legislation.

As for quality assessment, 82% of companies evaluate customer satisfaction only through a box for suggestions and complaints; moreover, 63% of aesthetic and beauty centers operate from Monday to Sunday, and the working hours that suits best for their needs was from 8:00 am to 7:00 pm de forma continua, since this was the response of 65% of study participants.

Of all businesses, 64% had less than 5 employees, 20% had more than 5 employees, and only 16% had more than 10 employees.

With regard to requirements for hiring employees, 48% of companies required a resume, a very low percentage if one considers the fact that many activities to be performed should require an academic background, which is relevant factor in view of the development of any activity that could pose high risks. It should also be noted that only 20% of companies required study certificates, and only 32% required criminal records, an indispensable requirement, since this type of company employs many people from Colombia’s neighboring countries.

As for the services provided, the most commonly provided service in aesthetic and beauty centers was hairdressing, with 21%, followed by manicure and pedicure, with 14%, and by make-up and waxing, with 12% and 13%, respectively. By summing these percentages, it was observed that 50% of respondents provide these services. Tanning and bodily treatments were the least commonly provided services, with 2% and 1%, respectively.

Conversely, 50% of centers had been selling beauty products; more specifically, the most commonly sold products were hair days, with 15%.

Furthermore, 47% of centers reported to perform invasive procedures, of which the most representative were eyebrow lift,^^17^^ perforations, tensor threads,^^18^^ and facial filling.^^19^^

It was observed that 44% of aesthetic and beauty centers received more than 15 clients a day, on average, while 36% reported to receive an average of less 10 clients a day; therefore, they could predict the number of clients per month and estimate an average flow of clients of at least 400 per month in 50% of aesthetic and beauty centers in Valledupar.

The most used tools in these centers were scissors, with 25%, followed by hairdryer and hair strengthener, with 24%. It was also found that 60% of study participants reported that clients spend in a beauty salon from 1 to 2 hours.

### LABOR AND HEALTH PROFILE OF WORKERS

With regard to the regimen that protects the workers of these companies, 61% were hired under a subsidized contract, and only 9% was a member of the four social insurance companies: risk administration company, healthcare insurance company, family compensation fund, and pension fund manager.

It is worth noting that 26% of employees performed administrative functions, 22% worked only as a stylist, 9% as an aesthetician, and the remaining participants perform other types de functions, often combining several roles, e.g., 13% of respondents performed manicure, pedicure, and administrative services.

Of workers, 71% performed the cleaning and hygiene of their working spaces, 50% reported having more than 5 years of experience in their function, 30% were technicians, 28% had a high school degree or a professional degree, and the majority (42%) were hired under a service provision contract.

It is worth noting that 93% of respondents are aware of biosafety protocols that should be implemented, none of respondents reported having a disability, 51% had a mean 2-hour rest during their working hours, and that 43% did not practice any sport.

Participants were asked on the occurrence of diseases like tinea, louse, herpes simplex, chickenpox, and cold. Of them, 97% reported they had a cold, 37% had protective items like anti-fluid uniform, mask, and gloves, and 97% did not have any type of health conditions in shoulders, wrists, or neck.

When asked about occupational accidents, 84% of respondents reported to have suffered an occupational accident in 2019, a concerning data considering the number of centers in Valledupar. Moreover, 77% of facilities did not have any work safety and health system, while only 8% of them did, and 31% of workers remain in the standing position from 1 to 2 hours.

### BIOSAFETY CONDITIONS

According to current legislation, aesthetic and beauty centers should have a space where all asepsis procedures should be performed. When asked about the subject, 55% of aesthetic and beauty centers reported not having a space for asepsis procedures; additionally, 91% did not perform any separation between clean and dirty equipment.

On one hand, 87% of centers did not disinfect nor sterilize equipment after serving a client; on the other, 77% did not disinfect tools other than metallic ones. With regard to items for storing tools, only 50% of centers used containers for storing sharp utensils.

With regard of control plans for cosmetic products and for disinfection products, it was found that only 33% and 30% of centers, respectively, had the documents to support each procedure to be conducted. Furthermore, 62% of centers had an appropriate ventilating system that were relevant to the activities they performed, and only 29% disinfected the floor. It is also worth highlighting that 49% of centers used disposable towels to dry clients’ hair, 72% did not have an organization and hygiene program, and 74% did not have a procedure for cleaning and disinfecting hands.

Results showed that 77% of centers did not complied with standards for cleaning and disinfection of tools other than metallic ones, and that the most used disinfectant was hypochlorite, which was used by 78% of centers. Furthermore, the most used sterilization methods were dry heat and saturated steam under pressure, with 41% and 34%, respectively.

As for waste separation, this was performed by only 50% of centers; moreover, 78% of centers did not have a space for food consumption.

Next, some variables from the different questionnaires are related to certain elements relevant for biosafety.

### RELATIONSHIP BETWEEN HYGIENE PROGRAM AND DISINFECTION OF ITEMS IN THE CENTER

An analysis of these two variables was performed to investigate the impact of an organization and hygiene program on cleaning procedures. It was found that 50% of respondents cleaned the lamps in centers with an organization and hygiene program; whereas 39% of participants working in centers with no such program performed the same procedure. This relationship was also observed with regard to cleaning the floor.

### RELATIONSHIP BETWEEN SPACE FOR ASEPSIS AND TOOL DISINFECTION

The importance of the space for asepsis in terms of biosafety relies of the fact that it is the place designed for disinfection of instruments and equipment. Therefore, the existence of this area implies that the center has been developing adequate protocols and complying with a legal requirement. With regard to the above relationship, it was found that, in centers with a space for asepsis, 38.1% of respondents disinfect cosmetology tools, 42.8% disinfect pedicure tools, and 4.76% disinfect hair tools. Conversely, in centers with no sterilization area, the percentage of workers who perform disinfection is the following: 73% for cosmetology tools, and 19.2% for pedicure ones.

### RELATIONSHIP BETWEEN WASTE SEPARATION AND PLACING OF SHARP ITEMS

With regard to waste separation, it was shown that 60% of respondents working at companies who perform waste separation reported having a container to place sharp items; conversely, only 31.8% of those working at companies with no such separation place sharp items into containers designed for this purpose.

APPROPRIATE VENTILATION AND INACTIVATION OF HAZARDOUS WASTE

Analysis of this relationship yielded the following results: in centers with appropriate ventilation, only 55.1% of respondents perform the inactivation of hazardous waste; however, in centers with no appropriate ventilation, 100% of respondents did not perform any inactivation of waste.

## DISCUSSION

The results from this research corroborate those reported in other studies, although some components showed some differences. It is also important to consider that comparison of results from the present study to current regulations identified major legal violations. The most representative study findings are described below.

The fact that 93% of companies are legally constituted represents a pivotal factor when creating improvement processes or assessment of compliance with legislation. Furthermore, the beauty sector is experiencing a growth rate;^^20^^ therefore, it is possible to implement strategies that, in one or another, promote improvements in biosafety processes.

The present study found that 83% of respondents are aware of legislation. With regard to this subject, López y Sandoval^^21^^ found that 50% of the population they assessed in Tulcán, Ecuador, was not aware of biosafety legislation and that 74% of companies had not implemented a protocol on the matter. These findings coincide with those of Santamaría et el.,^^22^^ who observed that 18.8% of tattoo centers in Tunja, Colombia, follow biosafety protocols, which may be a trend in these places, since another study^^21^^ shows that 69% of surveyed centers are not aware of biosafety protocols.

Furthermore, only 20% of centers established study requirements for hiring staff, which is a very low percentage, since the legislation^^11^^ states that this is a requirement for the good operating and opening of an aesthetic center. In their study, Bordin et al.^^23^^ revealed that 58.9% of the staff working at these centers did not complete high school, which show that the professionals working in the beauty industry are not developing processes to improve their skills to perform functions in connection with this industry.

It is important to emphasize that only 37% of respondents reported complying safety and health requirements concerning personal protection equipment in their workplace, an important condition for the operation of centers and that violate regulations on this matter, which establishes minimal safety and health standards in the workplace.^^24^^ Moreover, another study^^6^^ conducted within the beauty industry showed that 32% of respondents did not wear personal protective equipment.

## CONCLUSION

The analysis of biosafety conditions in aesthetic and beauty centers in Valledupar found that they are mostly legally constituted and are subjected to the legal operation legislation. Furthermore, workers at these centers are aware of biosafety regulations that should be adopted in order for the business to comply with appropriate procedures. However, beauty centers did not strictly comply with hiring requirements set forth by legislation.

The most commonly provided services are hairdressing, manicure, and pedicure, and the least common were bodily treatments and tanning. Moreover, nearly half of facilities perform invasive procedures, of which the most representative were eyebrow lifting, perforations, tensor threads, and facial filling, and the most used tools were scissors and hair strengtheners.

It was also found that client may spend an average of between 1 from 2 hours in an aesthetic and beauty center, and that these centers may receive an average of 400 clients per month, which is a significant, expressive number, in view of the risks posed for these people, in addition to possible transmission of diseases. With regard to workers’ safety and health, the picture is not very positive, since most employees are hired under a subsidized contract, approximately the half is hired under a service provision contract, and one fourth of them reported not being covered a safety and security program in their workplace.

With regard to security protocols as established by regulations, the present study showed that aesthetic centers have not been complying with requirements and are adopting inadequate procedures, which endangers not only workers’ health but also that of the clients, creating situations that may favor contamination processes and thus leads to diseases both in the working population and in visitors or clients.
